# Prognostic implications of N-terminal pro–B-type natriuretic peptide in patients undergoing transcatheter aortic valve implantation

**DOI:** 10.1093/ehjopen/oeaf169

**Published:** 2025-12-18

**Authors:** Nadia Salerno, Isabella Leo, Giovanni Canino, Antonio Bellantoni, Assunta Di Costanzo, Francesco Comito, Giuseppe Antonio Mazza, Giuseppe Panuccio, Salvatore Giordano, Salvatore De Rosa, Daniele Torella, Sabato Sorrentino

**Affiliations:** Department of Experimental and Clinical Medicine, Magna Graecia University of Catanzaro, 88100 Viale Europa, Catanzaro, Italy; Department of Experimental and Clinical Medicine, Magna Graecia University of Catanzaro, 88100 Viale Europa, Catanzaro, Italy; Department of Experimental and Clinical Medicine, Magna Graecia University of Catanzaro, 88100 Viale Europa, Catanzaro, Italy; Department of Medical and Surgical Sciences, Magna Graecia University of Catanzaro, 88100 Viale Europa, Catanzaro, Italy; Department of Medical and Surgical Sciences, Magna Graecia University of Catanzaro, 88100 Viale Europa, Catanzaro, Italy; Department of Experimental and Clinical Medicine, Magna Graecia University of Catanzaro, 88100 Viale Europa, Catanzaro, Italy; Department of Medical and Surgical Sciences, Magna Graecia University of Catanzaro, 88100 Viale Europa, Catanzaro, Italy; Department of Experimental and Clinical Medicine, Magna Graecia University of Catanzaro, 88100 Viale Europa, Catanzaro, Italy; Department of Experimental and Clinical Medicine, Magna Graecia University of Catanzaro, 88100 Viale Europa, Catanzaro, Italy; Department of Medical and Surgical Sciences, Magna Graecia University of Catanzaro, 88100 Viale Europa, Catanzaro, Italy; Department of Experimental and Clinical Medicine, Magna Graecia University of Catanzaro, 88100 Viale Europa, Catanzaro, Italy; Department of Medical and Surgical Sciences, Magna Graecia University of Catanzaro, 88100 Viale Europa, Catanzaro, Italy

**Keywords:** N-terminal pro–B-type natriuretic peptide, Transcatheter aortic valve implantation, Heart failure

## Abstract

**Aims:**

N-terminal pro–B-type natriuretic peptide (NT-proBNP) is a recognized marker of myocardial wall stress, but its prognostic role in patients undergoing transcatheter aortic valve implantation (TAVI) remains incompletely defined. This study assessed whether NT-proBNP levels at admission and discharge – interpreted using age-specific guideline thresholds – are associated with long-term clinical outcomes post-TAVI.

**Methods and results:**

We retrospectively analysed 683 consecutive patients who underwent successful TAVI at Magna Graecia University between 2009 and 2023. NT-proBNP was measured at both admission and discharge. Patients were stratified into low or high NT-proBNP groups based on age-adjusted cutoffs. Among 468 patients with paired measurements, four NT-proBNP trajectory groups were identified: Low–Low, Low–High, High–Low, and High–High. The primary endpoint was a composite of all-cause mortality or heart failure (HF) rehospitalization at 2 years. Multivariable Cox models were used to adjust for confounders. At admission, 41.6% of patients had elevated NT-proBNP, associated with worse echocardiographic parameters and more comorbidities. Elevated baseline NT-proBNP predicted a higher risk of the primary outcome (26.1% vs. 13.7%; HR 2.23; 95% CI, 1.51–3.28) and all-cause mortality (21.3% vs. 9.6%; HR 2.40; 95% CI, 1.52–3.79). Among patients with serial values, 34.6% had persistently elevated NT-proBNP, while only 10.7% improved. High–High and Low–High groups showed worse outcomes compared to Low–Low; High–Low patients had comparable risk to Low–Low.

**Conclusion:**

NT-proBNP, interpreted with age-specific thresholds, is a strong independent predictor of adverse outcomes after TAVI. Serial assessment adds prognostic value and may help guide postprocedural management.

## Introduction

Transcatheter aortic valve implantation (TAVI) has become the standard of care for patients with severe symptomatic aortic stenosis (AS), with its indications now extended across the entire spectrum of surgical risk.^1^ Despite these advances, substantial variability remains in both the timing of intervention and postprocedural outcomes, underscoring the need for robust tools to guide clinical decision-making throughout the care continuum.^2^

In this context, biomarkers capable of identifying patients at increased long-term risk may enhance follow-up strategies and inform therapeutic decisions.^3^ Among the most promising, N-terminal pro–B-type natriuretic peptide (NT-proBNP) is a well-established marker of myocardial wall stress and is widely used for both the diagnosis and prognostication of heart failure (HF).^4^ In AS, elevated NT-proBNP levels have been associated with symptom onset, disease severity, and adverse outcomes. Importantly, higher preprocedural NT-proBNP values have also been linked to increased mortality and HF-related rehospitalization following TAVI.^5–8^ Nevertheless, the optimal timing of measurement, appropriate threshold values, and the prognostic significance of both baseline levels and dynamic changes in NT-proBNP remain inadequately defined.

Recent European Society of Cardiology (ESC)-endorsed consensus statements have proposed age-adjusted NT-proBNP thresholds for the diagnosis of acute HF (≥450 pg/mL for patients <50 years, ≥900 pg/mL for those aged 50–75 years, and ≥1800pg/mL for those >75 years), demonstrating strong diagnostic accuracy in emergency settings. However, whether these thresholds can be extrapolated to risk stratification in the TAVI population has yet to be validated.^4^

In this study, we aimed to evaluate the prognostic value of NT-proBNP levels measured both before and after TAVI in a large real-world cohort, applying age-specific thresholds derived from current guidelines and expert consensus. This approach is intended to clarify the longitudinal prognostic utility of NT-proBNP in TAVI recipients and to support the integration of biomarker-guided strategies into routine postprocedural management.

## Methods

### Study population

Consecutive patients who underwent successful TAVI between September 2009 and December 2023 at the Cardiology Department of Magna Graecia University were retrospectively included in a pre-specified institutional dataset. All clinical data were collected from electronic medical records and anonymized before analysis. The study was conducted in accordance with the principles of the Declaration of Helsinki and approved by the local institutional review board.

### Study protocol and definitions

All procedures were performed according to standard clinical practice. Both balloon-expandable and self-expandable valves were used. The transfemoral approach was predominantly adopted, with percutaneous access and the use of a pre-closure technique.^9^ Prosthesis sizing was based on pre-procedural computed tomography. Rapid right ventricular pacing was routinely applied during balloon dilation for native or bioprosthetic valve deployment. Anaemia was defined per WHO sex-specific thresholds (Hb <13.0 g/dL men; <12.0 g/dL women); severity categories were mild 11.0–12.9/11.0–11.9 g/dL, moderate 8.0–10.9 g/dL, severe <8.0 g/dL. NT-proBNP levels were measured at admission and at discharge. NT-proBNP was quantified using the cobas h 232 NT-proBNP (Plus) assay (Roche Diagnostics); results are reported in pg/mL, with a uniform platform used across the study period. Clinical follow-up assessments were scheduled at 1 month, 6 months, and 2 years post-procedure. Decompensated heart failure was defined according to consensus-based, age-adjusted NT-proBNP thresholds: ≥450 pg/mL for patients <50 years, ≥900 pg/mL for those aged 50–75 years, and ≥1800pg/mL for patients >75 years.^4^ Patients with values above these thresholds were classified as having elevated NT-proBNP levels. Additionally, patients were categorized into four NT-proBNP trajectory groups based on pre- and post-procedural values using the guideline-recommended cutoffs previously reported: (1) Low–Low: NT-proBNP below threshold at both admission and discharge; (2) Low–High: NT-proBNP below threshold at admission, but above threshold at discharge; (3) High–Low: NT-proBNP above threshold at admission, but below threshold at discharge; (4) High–High: NT-proBNP above threshold at both time points. To assess potential selection from missing trajectories, we compared baseline characteristics of patients with vs. without serial NT-proBNP. The primary outcome measure was a composite of all-cause mortality or HF rehospitalization at 2 years of follow-up. Secondary outcomes were all-cause mortality at 2 years of follow-up and in-hospital bleeding, pacemaker implantation, and new-onset atrial fibrillation. All the outcomes were defined according to the Valve Academic Research Consortium 3 consensus.

### Statistical analysis

Continuous variables were expressed as mean ± standard deviation and compared using Student’s *t*-test. Categorical variables were expressed as frequencies and percentages and compared between groups using the chi-squared test. Event rates were estimated using the Kaplan–Meier method and compared with the log-rank test. The primary endpoint was analysed using multivariable Cox proportional hazards regression to estimate the adjusted risk of death or HF rehospitalization associated with elevated NT-proBNP levels, as previously defined. Covariates included in the multivariable model were selected based on baseline imbalances and established prognostic relevance in the literature. For sensitivity, we repeated the Cox analyses within atrial fibrillation (AF) strata and by procedural period – early (2009–2016) vs. contemporary (2017–2023). Within each stratum, we ran (i) a univariable Cox with high NT-proBNP at admission as the exposure and (ii) a multivariable Cox model. We also fit a unified model with an NT-proBNP × period interaction. As an additional sensitivity analysis, we built a parsimonious model that included EuroSCORE II (entered per 1% absolute risk) together with NT-proBNP, AF, BMI, anaemia, smoking, and dyslipidaemia. The discriminative performance of the Cox regression models was assessed using Harrell’s concordance statistic (C-statistic), which quantifies the model’s ability to correctly rank the survival times of patients. A C-statistic value of 0.5 indicates no discriminative power, while a value of 1.0 indicates perfect discrimination.

Statistical analyses were performed using Stata version 12.1 (StataCorp, College Station, TX, USA). A two-tailed *P*-value <0.05 was considered statistically significant.

## Results

### Baseline and echocardiographic characteristics

**Figure 1:**
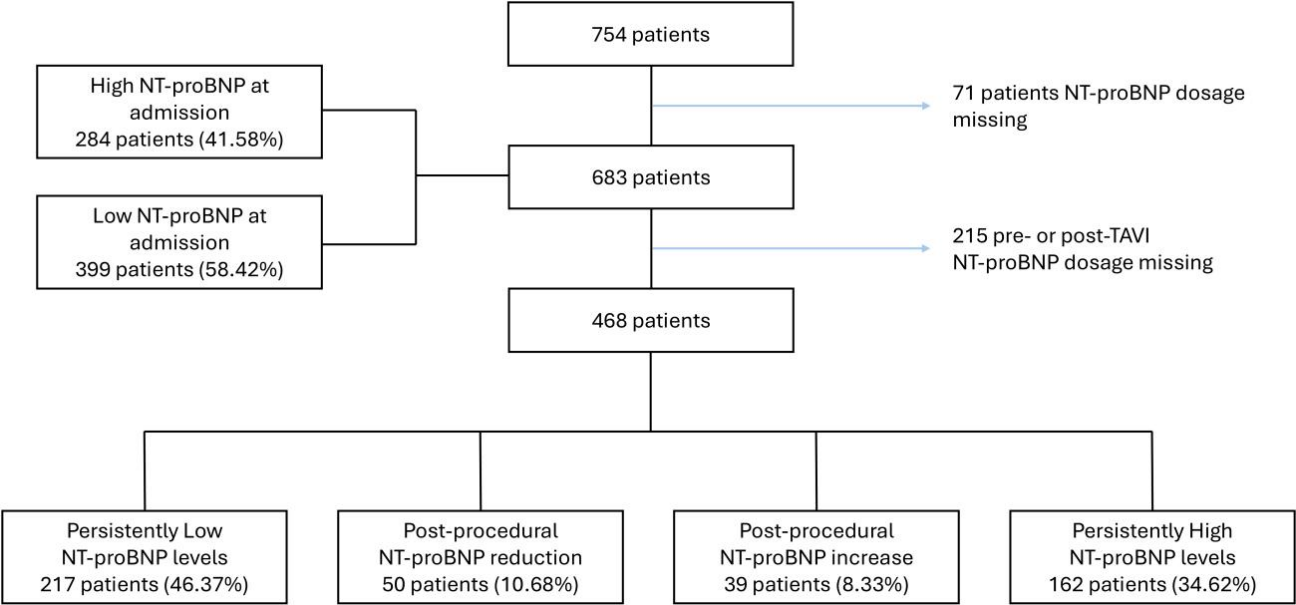
Study flow chart.

A total of 683 patients with severe AS undergoing TAVI were included in the analysis (*Figure 1* and Supplementary material online, *Table S1*). Among them, 284 patients (41.6%) presented with elevated NT-proBNP levels at admission, as defined by prespecified age-adjusted thresholds. Baseline characteristics stratified by NT-proBNP levels are summarized in *Table 1*.

**Table 1:** Baseline clinical characteristics according to NT-proBNP levels at admission based on guideline-recommended cutoffs

Variables	Low NT-proBNP (*n* = 399)	High NT-proBNP (*n* = 284)	*P* value
Age	80.12 ± 5.30	80.14 ± 6.36	0.52
Female sex	221 (55.39)	156 (54.93)	0.95
BMI, kg/m²	28.13 ± 4.77	26.90 ± 4.65	<0.001
BSA, m²	1.80 ± 0.18	1.76 ± 0.20	0.005
NYHA Class III/IV	247 (76.47)	218 (90.08)	<0.001
Euroscore II	4.45 ± 4.10	5.98 ± 5.07	<0.001
Hypertension	376 (94.24)	255 (89.79)	0.03
Dyslipidaemia	290 (72.68)	170 (59.86)	<0.001
Diabetes Mellitus type 2	140 (35.09)	105 (36.97)	0.61
Smoking history	86 (21.61)	45 (15.85)	0.06
Prior cerebrovascular event	36 (9.02)	24 (8.45)	0.80
Prior Valvular Surgery	16 (3.91)	12 (4.38)	0.67
Prior PCI	85 (21.30)	62 (21.83)	0.87
Chronic kidney disease	81 (19.80)	100 (36.50)	<0.001
Severe Chronic kidney disease^a^	11 (2.78)	62 (22.06)	<0.001
Atrial Fibrillation	69 (17.29)	99 (34.86)	<0.001
Prior pacemaker implantation	28 (7.02)	26 (9.15)	0.31
Peripheral artery disease	60 (15.04)	33 (11.62)	0.20
COPD	88 (22.06)	60 (21.13)	0.77
Haemoglobin, g/dL	12.44 (1.60)	11.90 (1.68)	<0.001
Creatinine, mg/dL	1.04 (0.55)	1.56 (1.51)	<0.001
Platelets, 10³/μL	216 (71.54)	203 (70.78)	0.01
**Baseline Echo Variables**			
LVEF	54.96 ± 5.27	48.15 ± 9.37	<0.001
LVEF < 50%	60 (15.04)	153 (53.87)	<0.001
Mean Gradient, mmHg	46.40 ± 11.80	51.35± 16.01	<0.001
Peak Gradient, mmHg	72.15± 19.52	75.70± 26.45	0.03
Aortic Valve area baseline, cm^2^	0.77 ± 0.15	0.67 ± 0.18	<0.001
PAPS, mmHg	37.85 ± 8.91	44.34 ± 12.66	<0.001
TAPSE, mm	22.74 ± 2.68	21.32 ± 3.54	<0.001
Moderate or Severe MR	24 (6.02)	50 (17.73)	<0.001
Moderate or Severe TR	19 (4.76)	29 (10.39)	0.005
Moderate or Severe AR	19 (4.76)	29 (10.39)	0.005

Data are reported as mean ± standard deviation (SD) or as number and (percentage).

COPD, Chronic Obstructive Pulmonary Disease; PAPs, Pulmonary artery systolic pressure; TAPSE, Tricuspid Annular Plane Systolic Excursion; LVEF, Left ventricular ejection fraction; MR, mitral regurgitation. TR tricuspid regurgitation; AR, aortic regurgitation.

^a^< 30 mL/min/1.73 m².

There were no significant differences between the two groups in terms of age, sex, diabetes, or prior cardiovascular interventions. However, patients with elevated NT-proBNP levels exhibited significantly lower body mass index (BMI) and body surface area (BSA), and more frequently presented with NYHA class III–IV symptoms (*P* < 0.01). The high NT-proBNP group also had a significantly greater prevalence of chronic kidney disease (CKD), and atrial fibrillation (AF) (*P* < 0.001 for all).

Echocardiographic assessment revealed worse baseline cardiac morphology and function in the high NT-proBNP group, including lower left ventricular ejection fraction (LVEF), smaller aortic valve area, higher transvalvular gradients, elevated pulmonary artery pressures (PAPs), and a higher prevalence of both right and left valvular dysfunction (*P* < 0.01 for all comparisons). Laboratory evaluation showed significantly lower haemoglobin and platelet counts, and higher serum creatinine concentrations in patients with elevated NT-proBNP levels (*P* < 0.01 for all).

### Preprocedural and postprocedural NT-proBNP distribution

**Figure 2:**
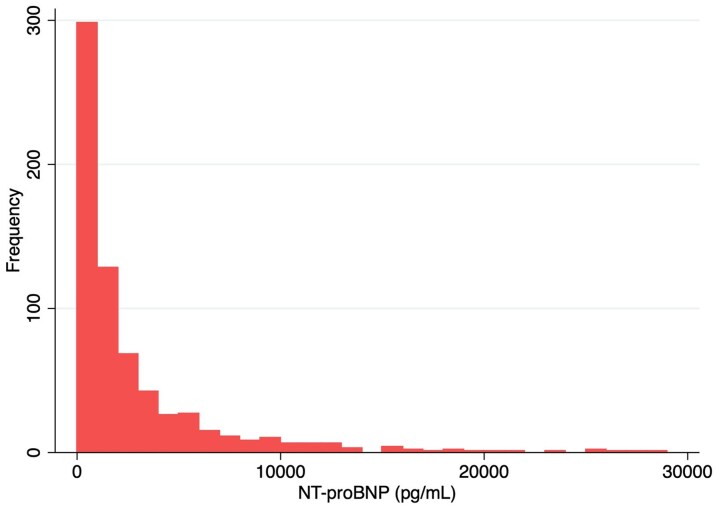
NT-proBNP distribution at admission.

*Figure 2* illustrated the distribution of NT-proBNP levels at admission. The median NT-proBNP level was 1219 pg/mL (IQR: 513–3457 pg/mL), while the mean was substantially higher at 3391 pg/mL due to the influence of outliers.

**Figure 3:**
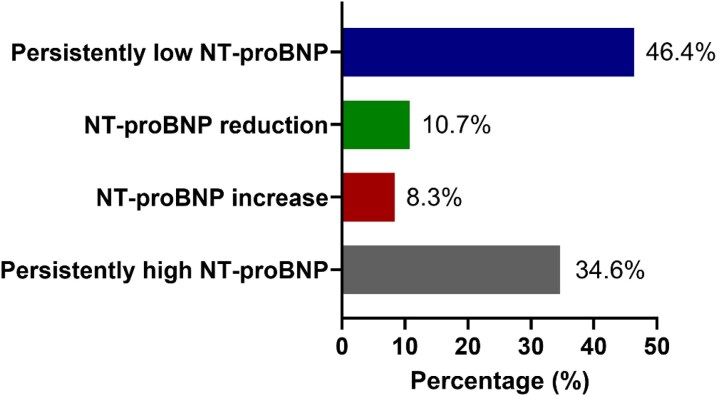
Distribution of NT-proBNP patterns.

Based on guideline-recommended age-adjusted thresholds (*Table 2*), 51 patients (17.96%) aged 50–74 years and 233 patients (82.04%) aged ≥75 years had elevated NT-proBNP levels at baseline. Among the 683 patients included, discharge NT-proBNP measurements were available for 468 individuals. Clinical and procedural characteristics of patients without available NT-proBNP trajectories are presented in Supplementary material online, *Table S2* and did not substantially differ from those of the remaining study population. Of these, 217 patients (46.4%) exhibited persistently low NT-proBNP levels, 50 (10.7%) showed a postprocedural reduction, 39 (8.3%) had a postprocedural increase, and 162 (34.6%) had persistently elevated NT-proBNP levels (*Figure 3*).

**Table 2:** Pre-procedural NT-proBNP distribution according to guideline-recommended age-specific thresholds

Age category	Guideline-recommended NT-proBNP cutoff	Patients with elevated NT-proBNP, *n* (%)	NT-proBNP concentration pg/mL (mean; IQR)
Age < 50 years	450 pg/mL	–	–
Age 50–74 years	900 pg/mL	51 (17.96%)	879.00 (430.00–3385.00)
Age ≥ 75 years	1800 pg/mL	233 (82.04%)	1281.50 (534.00–3457.00)

Data are reported as mean ± interquartile range or as number and (percentage).

### Procedural and clinical outcomes

Procedural characteristics and in-hospital outcomes stratified by NT-proBNP levels are reported in *Table 3*. Patients with elevated NT-proBNP at admission experienced significantly longer lengths of stay in the Coronary Care Unit (CCU) compared to those with low levels (*P* < 0.001). No significant differences were observed in procedural characteristics or the incidence of periprocedural complications.

**Table 3:** Procedural and in-hospital outcomes according to NT-proBNP levels at admission based on guideline-recommended cu-offs

Variables	Low NT-proBNP (*n* = 399)	High NT-proBNP (*n* = 284)	*P* value
CCU Stay, h	65.41 ± 48.94	83.80 ± 95.98	<0.001
Fluoro Time, min	21.81 ± 10.61	23.26 ± 12.27	0.07
Valve in valve	10 (2.51)	9 (3.17)	0.60
Self-expandable valve	284 (71.18)	220 (77.46)	0.07
Death IH	4 (1.00)	8 (2.82)	0.08
Major bleeding	44 (11.06)	37 (13.03)	0.43
Minor bleeding	32 (9.94)	34 (13.99)	0.14
Pacemaker implantation			
PM	69 (17.34)	55 (19.37)	0.08
ICD	3 (0.75)	8 (2.82)	0.08
New onset AF	30 (7.52)	26 (9.15)	0.44
**Discharge Medications**			
Aspirin	278 (70.03)	166 (58.66)	0.002
P2Y12 inhibitor	230 (57.93)	160 (56.54)	0.72
Anticoagulants	105 (26.32)	118 (41.55)	<0.001
Beta blockers	265 (67.26)	189 (68.23)	0.79
Calcium Blocker	68 (17.26)	55 (19.86)	0.39
ACE-I	169 (42.89)	127 (46.01)	0.42
ARBS	194 (49.24)	88 (31.88)	<0.001
MRA	44 (11.17)	85 (30.80)	<0.001
Diuretics	296 (75.51)	240 (86.96)	<0.001
ARNI	6 (1.55)	10 (3.72)	<0.001
**ECHO post**			
LVEF	54.59 ± 5.41	48.51 ± 9.00	<0.001
Mean gradient post	10.10 ± 4.89	10.58 ± 6.02	0.12
Aortic Regurgitation 2–3+	19 (4.76)	29 (10.39)	0.005
PAPS, mmHg	37.07 ± 8.70	41.06 ± 10.41	<0.001
TAPSE, mm	22.93 ± 2.49	21.99 ± 3.30	<0.001

CCU, Coronary Care Unit; PM, Pacemaker; ICD, Implantable Cardioverter-Defibrillator; AF, Atrial Fibrillation; ACE-I, angiotensin-converting enzyme inhibitor; ARBS, Angiotensin II Receptor Blockers; MRA, Mineralocorticoid Receptor Antagonist; ARNI, Angiotensin Receptor–Neprilysin Inhibitor; LVEF, Left ventricular ejection fraction; PAPs, Pulmonary artery systolic pressure; TAPSE, Tricuspid Annular Plane Systolic Excursion.

Postprocedural echocardiographic evaluations demonstrated that patients in the high NT-proBNP group continued to have significantly lower LVEF, higher PAPs, and reduced TAPSE (*P* < 0.001 for all). Moderate-to-severe postprocedural aortic regurgitation was more frequent in this group (*P* = 0.005), while mean transvalvular gradients were comparable between groups (*P* = 0.12).

**Figure 4:**
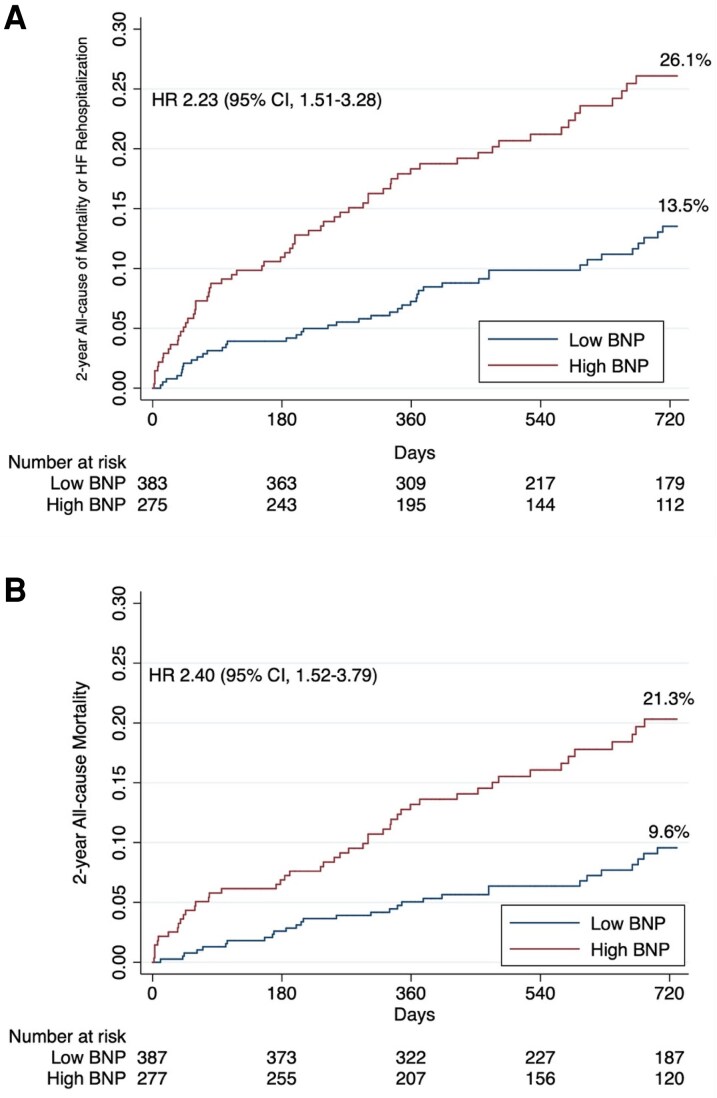
(*A*) Risk of all-cause mortality and HF rehospitalization at 2 years of follow-up according to NT-proBNP levels at admission based on guideline-recommended cutoffs. (*B*) Risk of all-cause mortality at 2 years of follow-up according to NT-proBNP levels at admission based on guideline-recommended cutoffs.

Over a 2-year follow-up, patients with elevated NT-proBNP levels at baseline experienced a significantly higher incidence of the composite endpoint of all-cause mortality or HF rehospitalization compared to those with low baseline levels (26.1% vs. 13.7%; HR 2.23, 95% CI 1.51–3.28; *Figure 4A*). Similarly, all-cause mortality alone was higher in the high NT-proBNP group (21.3% vs. 9.6%; HR 2.40, 95% CI 1.52–3.79; *Figure 4B*). The predictive performance of NT-proBNP for these outcomes was modest, with C-statistics of 0.613 and 0.606, respectively. Importantly, elevated preprocedural NT-proBNP remained an independent predictor of the primary outcome after multivariable adjustment (HR 1.85; 95% CI 1.18–2.88; *P* = 0.007; *Table 4*). Sensitivity analyses confirmed prognostic value across AF status and implantation period, with no significant interactions (see Supplementary material online, *Table S3*, Panel A-B). In an additional model incorporating EuroSCORE II as a global surgical-risk measure, high NT-proBNP at admission remained independently associated with the 2-year endpoint (HR 1.88, 95% CI 1.22–2.89; *P* = 0.004) (see Supplementary material online, *Table S4*).

**Table 4:** Multivariable Cox regression analysis for 2-year all-cause mortality or heart failure rehospitalizzation

Variable	Hazard ratio	95% confidence interval	*P*-value
High NT-proBNP at admission	1.85	1.18–2.88	0.007
Female sex	1.30	0.85–2.00	0.231
BMI (per unit)	0.99	0.95–1.04	0.802
Anaemia	1.31	0.88–1.96	0.185
Smoking	1.73	1.05–2.84	0.030
Baseline LVEF (per %)	0.99	0.96–1.01	0.239
COPD	1.65	1.07–2.55	0.023
Dyslipidaemia	0.72	0.48–1.07	0.104
Atrial Fibrillation	1.45	0.96–2.21	0.078
Chronic Kidney Disease	0.99	0.64–1.52	0.949

BMI, Body Mass Index; NT-proBNP, N-terminal pro-B-type natriuretic peptide; LVEF, Left Ventricular Ejection Fraction; COPD, Chronic Obstructive Pulmonary Disease.

**Figure 5:**
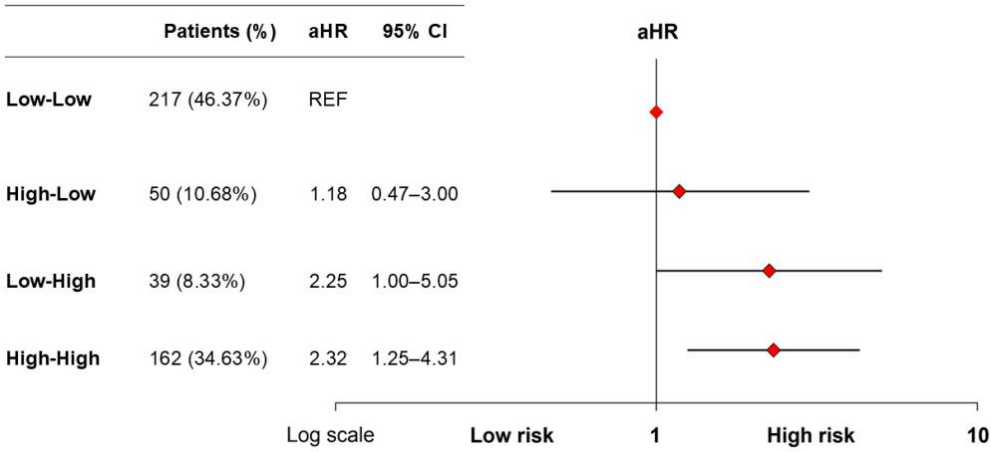
Risk of 2-year mortality or HF rehospitalization according to periprocedural NT-proBNP trajectories.

Finally, compared to patients with persistently low NT-proBNP levels, no significant difference in the primary outcome was observed in those who exhibited a postprocedural reduction in NT-proBNP (aHR 1.18; 95% CI 0.47–3.00), while those with postprocedural NT-proBNP elevation (aHR 2.25; 95% CI 1.00–5.05) and those with persistently high levels (aHR 2.32; 95% CI 1.25–4.31) had significantly greater risk (*Figure 5*), based on models adjusted for sex, BMI, anaemia, LVEF, COPD, dyslipidaemia, atrial fibrillation, and chronic kidney disease (*Table 5*). Results were consistent when EuroSCORE II was incorporated into the multivariable Cox model, with persistently high and increasing NT-proBNP trajectories remaining significantly associated with adverse outcomes, in line with the findings of the main analysis (see Supplementary material online, *Table S5*).

**Table 5:** Association between postprocedural NT-proBNP trajectories and 2-year risk of all-cause of mortality or heart failure rehospitalization

Variable	Hazard ratio	95% confidence interval	*P*-value
Post-procedural NT-proBNP reduction	1.18	0.47–3.00	0.723
Post-procedural NT-proBNP increase	2.25	1.00–5.05	0.049
Persistently high NT-proBNP levels	2.32	1.25–4.31	0.008
Female sex	1.02	0.64–1.63	0.941
BMI (per unit)	1.00	0.95–1.05	0.930
Anaemia	1.45	0.89–2.37	0.134
Baseline LVEF (per %)	0.99	0.96–1.02	0.513
COPD	2.27	1.40–3.67	0.001
Dyslipidaemia	0.73	0.46–1.16	0.181
Atrial Fibrillation	1.33	0.82–2.16	0.250
Chronic Kidney Disease	0.90	0.54–1.48	0.675

NT-proBNP, N-terminal pro-B-type natriuretic peptide; BMI, Body Mass Index; LVEF, Left Ventricular Ejection Fraction; COPD, Chronic Obstructive Pulmonary Disease.

## Discussion

In this retrospective cohort of nearly 700 patients undergoing TAVI, we evaluated the prognostic utility of NT-proBNP levels measured both before and after the procedure. The main findings emanating from our study are as follows: (i) Preprocedural NT-proBNP levels exhibited a markedly right-skewed distribution, reflecting substantial heterogeneity in disease severity and systemic stress; (ii) Approximately 50% of patients exceeded the age-specific thresholds recommended by ESC consensus statements for the diagnosis of acute decompensated heart failure (HF); (iii) Elevated baseline NT-proBNP levels were independently associated with a two-fold increase in the risk of all-cause mortality or HF rehospitalization at 2 years; (iv) Age-adjusted, consensus-based cutoffs showed fair discriminative capacity for predicting long-term adverse outcomes; and (v) Persistently elevated or rising NT-proBNP levels post-TAVI identified patients at particularly high risk, whereas a postprocedural reduction in NT-proBNP was associated with improved prognosis.

NT-proBNP is a well-established biomarker for risk stratification in patients with AS, with prognostic value in both symptomatic and asymptomatic stages.^10^ It has been increasingly incorporated into longitudinal surveillance strategies aimed at detecting early signs of disease progression and decompensation.^11^ Notably, in the multicentre, randomized EARLY TAVR trial, NT-proBNP was used as part of a predefined algorithm to trigger early intervention. Specifically, a threefold rise from baseline – alongside an absolute NT-proBNP value >375 pg/mL in patients ≤75 years or >1350 pg/mL in those >75 years – was used to prompt aortic valve replacement. Importantly, nearly 40% of patients in the surveillance arm developed overt symptoms during follow-up, highlighting the limitations of symptom-based monitoring alone.^12^

Despite these promising applications, the broader integration of NT-proBNP into clinical decision-making remains hindered by the absence of standardized protocols regarding its timing, interpretation, and role across different disease stages. For instance, the 2021 ESC Guidelines for Valvular Heart Disease recommend natriuretic peptide measurement only in select asymptomatic patients with preserved ejection fraction – underscoring a conservative approach that positions NT-proBNP as a secondary, rather than primary, clinical tool.^1^

Nevertheless, consistent evidence from large-scale observational studies supports its robust prognostic value. Medranda et al., for example, reported that baseline BNP levels ≥500 pg/mL were associated with a > 3-fold increased risk of stroke or death at 30 days post-TAVI.^7^ Similarly, Sørensen et al. demonstrated that NT-proBNP ≥420 pmol/L independently predicted a twofold higher risk of one-year HF hospitalization and all-cause mortality.^13^

The variation in NT-proBNP thresholds across studies reflects differences in population characteristics, assay platforms, and clinical context – diagnostic vs. prognostic.^14^ In our study, we minimized such variability by adopting the age-adjusted NT-proBNP cutoffs recommended by international consensus statements for acute HF diagnosis. These thresholds, originally derived from the ICON and ICON-RELOADED studies, have demonstrated consistent diagnostic accuracy across a range of clinical conditions and patient subgroups – irrespective of renal function, BMI, AF, or diabetes.^15,16^ Their application in our TAVI cohort yielded fair predictive value, reinforcing their broader potential in risk stratification beyond acute care settings.

Importantly, nearly half of our TAVI patients met the NT-proBNP criteria for acute HF despite being referred for elective intervention. This finding underscores the limitations of symptom-based referral pathways and suggests that a substantial proportion of high-risk patients may experience delays in treatment. Our results advocate for a more structured, biomarker-guided surveillance approach, particularly in elderly, frail, or minimally symptomatic individuals, in whom clinical evaluation may underestimate disease burden.

Beyond static measurements, our analysis also explored NT-proBNP trajectories surrounding the procedure. Patients who demonstrated postprocedural reductions – i.e. ‘biochemical responders’ – experienced significantly lower rates of death or HF rehospitalization. These findings align with those of Seoudy et al., who similarly reported improved outcomes in patients with declining NT-proBNP levels at discharge.^6^ Interestingly, while responders typically presented with more severe preprocedural haemodynamic compromise, non-responders exhibited a higher burden of comorbidities – suggesting distinct pathophysiologic substrates underlying differential NT-proBNP dynamics. Indeed, from a mechanistic standpoint, persistently elevated or rising NT-proBNP levels after TAVI likely reflect unresolved myocardial stress and maladaptive cardiac remodelling. Although relief of valvular obstruction by TAVI reduces left ventricular afterload, many patients – particularly those with long-standing pressure overload – may already exhibit irreversible structural changes, including myocardial fibrosis, impaired diastolic function, or reduced contractile reserve. These pathophysiologic alterations can blunt the haemodynamic benefits of valve replacement and sustain neurohormonal activation, thereby maintaining high natriuretic peptide levels.^17^ Comorbidities like AF, renal dysfunction, and pulmonary hypertension may further exacerbate myocardial load.^18,19^ In contrast, patients who exhibit postprocedural NT-proBNP decline may represent a subgroup with more favourable myocardial substrate and greater capacity for reverse remodeling.^5,6^

Taken together, these findings highlight the value of NT-proBNP not only as a static prognostic biomarker but also as a dynamic tool for postprocedural risk stratification. This may be particularly relevant in the context of emerging cardioprotective therapies – such as Sodium-glucose cotransporter-2 inhibitors (SGLT2i) and angiotensin receptor–neprilysin inhibitors (ARNI) – which have demonstrated benefits in selected high-risk populations.^20^ Early identification of such patients through NT-proBNP monitoring could enable the timely initiation of adjunctive medical therapy, ultimately improving long-term outcomes. This may be particularly relevant in light of the recently published DapaTAVI randomized trial, which demonstrated that dapagliflozin led to a 28% relative reduction in the composite endpoint of all-cause mortality or worsening HF, and a 37% reduction in HF events compared to standard care.^21^ Importantly, our patient cohort was enrolled before the availability of this evidence, highlighting a key opportunity for NT-proBNP–guided identification of high-risk individuals – such as those with persistently elevated levels – who may derive the greatest benefit from early initiation of SGLT2 inhibition. Such a strategy might have prevented HF progression and improved outcomes in our population.

### Limitations

This study has several limitations that warrant consideration. First, it is a retrospective, single-centre analysis in a TAVI-treated cohort, which may limit the external validity of our findings and carries inherent risks of selection and information bias despite multivariable adjustment. The absence of a comparator group of asymptomatic or less-severe AS patients managed without AVR/TAVI prevents any inference on biomarker-guided timing of intervention, and subgroup sample sizes were relatively small, limiting statistical power. Second, NT-proBNP assessment was based on routine clinical care, with variability in timing and some missingness. We relied on a single predischarge measurement for feasibility, while alternative post-TAVI time points and serial sampling beyond discharge were not systematically collected. As a result, we cannot establish the optimal timing of NT-proBNP measurement, and the discharge landmark may have introduced survivor bias. Third, although multivariable models adjusted for key clinical covariates, residual confounding cannot be excluded. Baseline LVEF was imbalanced, with many patients <50%, which may confound the association between NT-proBNP and outcomes. We mitigated this by modelling LVEF as a continuous covariate in all multivariable analyses (avoiding arbitrary cutoffs), but residual confounding cannot be excluded, so findings are prognostic rather than causal. Additional unmeasured factors – including frailty, functional status, inflammatory markers, and comorbidity burden – were not captured, and although EuroSCORE II was incorporated as a replacement composite to mitigate double-counting, it only partially reflects frailty and reserve capacity. Fourth, this study was not designed or powered to compare NT-proBNP with other candidate biomarkers or multimarker panels. Alternative markers such as hs-troponin, sST2, GDF-15, or galectin-3 were not collected systematically, and the prognostic discrimination of NT-proBNP was modest. The question of the most effective biomarker for treatment-timing or management decisions, therefore, remains open and warrants prospective, head-to-head comparative studies with decision-analytic metrics. Finally, patient selection may have led to conservative bias. The exclusion of less decompensated patients – characterized by lower NT-proBNP levels, higher LVEF/TAPSE, and lower PAPs – would likely bias associations toward the null. In addition, STS score, formal frailty indices, and health-status measures were not systematically collected. Although we adjusted for EuroSCORE II – entered as a replacement composite to avoid double-counting – this score only partially captures frailty and functional reserve, so residual confounding may persist. Collectively, these limitations underscore that our results should be interpreted as associative and hypothesis-generating rather than causal, and future prospective studies are warranted to validate and extend these findings.

## Conclusion

In this large, real-world cohort of patients undergoing TAVI, elevated NT-proBNP levels – defined according to age-specific ESC consensus thresholds – were independently associated with an increased risk of long-term mortality and HF rehospitalization. Patients with persistently elevated or rising NT-proBNP levels after TAVI experienced the poorest outcomes, whereas a postprocedural decline identified a subgroup with a more favourable prognosis. These findings support the role of NT-proBNP as a practical and informative biomarker for risk stratification both before and after TAVI. Integration of NT-proBNP monitoring into standardized clinical pathways may enhance patient selection, inform follow-up strategies, and guide timely initiation of emerging medical therapies aimed at improving post-TAVI outcomes.

## Supplementary Material

oeaf169_Supplementary_Data

## Data Availability

The data underlying this article will be shared on reasonable request to the corresponding author.
