# Supplementary material for: Prognostic implications of N-terminal pro–B-type natriuretic peptide in patients undergoing transcatheter aortic valve implantation
Source: Eur Heart J Open. 2025 Dec 18;6(1):oeaf169. doi: 10.1093/ehjopen/oeaf169 (PMC12768887; doi:10.1093/ehjopen/oeaf169)
Supplement: oeaf169_Supplementary_Data [file oeaf169_supplementary_data.docx]

**Supplementary Data**

**Supplementary Table S1. Study flow from all TAVI recipients to the cohort with serial NT-proBNP (admission + predischarge).**

| Step | N remaining | Excluded (n) | Reason(s) / criteria |
| --- | --- | --- | --- |
| All TAVI recipients screened | 754 |  | Initial cohort |
| With admission NT-proBNP within prespecified window | 683 | 71 | No admission NT-proBNP due to logistical/operational issues (e.g., off-hour admission, sample handling)  **Primary analysis cohort** |
| With predischarge NT-proBNP within prespecified window [within 24 h to discharge] | 468 | 215 | No predischarge NT-proBNP:   - logistical/operational issues (e.g., off-hour admission, sample handling) (n=203) - In hospital death (n=12) |
| Final analysis set with serial NT-proBNP (admission + predischarge) | 468 | — | **Secondary analysis cohort** |

Logistical/operational issues: Off-hour admission; sample handling; lab downtime; administrative issues. NT-proBNP: N-terminal pro–B-type natriuretic peptide

**Supplementary Table S2. Baseline characteristics by availability of NT-proBNP trajectory (Available vs Missing)**

| **Variables** | **Available (n=468)** | **Missing (n=215)** | **P value** |
| --- | --- | --- | --- |
| **Demographics & Laboratory** | | | |
| Age, years | 80.18 ± 5.75 | 80.02 ± 5.80 | 0.742 |
| Female sex, n (%) | 268 (57.26%) | 109 (50.70%) | 0.109 |
| BMI, kg/m² | 27.49 ± 4.52 | 27.91 ± 4.93 | 0.278 |
| BSA, m² | 1.78 ± 0.18 | 1.78 ± 0.19 | 0.88 |
| NYHA class III/IV, n (%) | 354 (83.89%) | 111 (77.62%) | 0.090 |
| EuroSCORE II | 5.29 ± 4.40 | 4.63 ± 4.94 | 0.090 |
| Hypertension, n (%) | 430 (91.88%) | 201 (93.49%) | 0.462 |
| Dyslipidemia, n (%) | 300 (64.10%) | 160 (74.42%) | 0.008 |
| Diabetes Mellitus type 2, n (%) | 172 (36.75%) | 73 (33.95%) | 0.479 |
| Smoking history, n (%) | 89 (19.02%) | 42 (19.63%) | 0.851 |
| Prior cerebrovascular event, n (%) | 38 (8.12%) | 22 (10.23%) | 0.365 |
| Prior valvular surgery, n (%) | 21 (4.49%) | 7 (3.26%) | 0.195 |
| Prior PCI, n (%) | 98 (20.94%) | 49 (22.79%) | 0.585 |
| Chronic kidney disease, n (%) | 133 (28.42%) | 48 (22.33%) | 0.094 |
| Severe Chronic kidney disease* | 55 (11.75%) | 18 (7.94%) | 0.133 |
| Atrial fibrillation, n (%) | 124 (26.50%) | 44 (20.47%) | 0.089 |
| Prior pacemaker/ICD implantation, n (%) | 34 (7.26%) | 20 (9.30%) | 0.359 |
| Peripheral artery disease, n (%) | 59 (12.61%) | 34 (15.81%) | 0.256 |
| COPD, n (%) | 99 (21.15%) | 49 (22.79%) | 0.630 |
| Hemoglobin, g/dL | 12.13 ± 1.67 | 12.40 ± 1.60 | 0.043 |
| Creatinine, mg/dL | 1.29 ± 1.16 | 1.18 ± 0.92 | 0.216 |
| NT-proBNP at admission, pg/mL | 3816.31 ± 7312.05 | 2465.54 ± 4430.26 | 0.013 |
| Platelets, 10³/μL | 212.67 ± 69.83 | 207.28 ± 74.72 | 0.362 |
| **Echocardiography** | | | |
| Baseline LVEF, % | 51.63 ± 8.35 | 53.21 ± 7.06 | 0.017 |
| LVEF < 50% |  |  |  |
| Baseline mean gradient, mmHg | 48.22 ± 13.27 | 48.96 ± 15.23 | 0.524 |
| Baseline peak gradient, mmHg | 72.49 ± 23.26 | 76.08 ± 21.18 | 0.071 |
| Aortic Valve area baseline, cm2 | 0.72 ±0.16 | 0.74±0.19 | 0.31 |
| PAPs, mmHg | 41.44 ± 11.44 | 38.65 ± 10.09 | 0.003 |
| TAPSE, mm | 21.94 ± 3.10 | 22.65 ± 3.12 | 0.011 |
| Moderate/severe MR, n (%) | 53 (11.37%) | 21 (9.77%) | 0.531 |
| Moderate/severe TR, n (%) | 35 (7.54%) | 13 (6.07%) | 0.488 |
| Moderate or Severe AR | 36 (7.76%) | 12 (5.61%) | 0.310 |
| Post-procedural peak gradient, mmHg | 18.86 ± 8.81 | 20.91 ± 9.64 | 0.177 |
| **Procedural & In-hospital outcomes** | | | |
| CCU stay, hours | 77.97 ± 80.32 | 62.00 ± 49.77 | 0.009 |
| Fluoroscopy time, min | 22.31 ± 11.39 | 22.51 ± 11.14 | 0.836 |
| Valve in valve | 14 (2.99%) | 5 (2.33%) | 0.623 |
| Self-expandable valve | 352 (75.21%) | 152 (70.70%) | 0.213 |
| In-hospital death, n (%) | 8 (1.71%) | 4 (1.86%) | 0.889 |
| Pacemaker/ICD implantation in-hospital, n (%) | 82 (17.52%) | 53 (24.77%) | 0.079 |
| Major bleeding, n (%) | 62 (13.25%) | 19 (8.88%) | 0.102 |
| Major access complication, n (%) | 42 (8.97%) | 17 (7.91%) | 0.645 |
| Minor bleeding, n (%) | 51 (12.11%) | 15 (10.42%) | 0.584 |

**Supplementary Table S3.** Sensitivity analyses by atrial fibrillation (AF) status and procedural period,

**Panel A.** Atrial fibrillation sensitivity analysis

| **Stratum** | **Model** | **Hazard Ratio** | **95% CI** | **p-value** | **Harrell’s C; PH global p** |
| --- | --- | --- | --- | --- | --- |
| Non-AF | Univariable | 2.30 | 1.43–3.70 | 0.001 | — |
| Non-AF | Multivariable | 1.79 | 1.06–3.05 | 0.031 | C=0.648; PH p=0.305 |
| AF | Univariable | 1.60 | 0.81–3.15 | 0.176 | — |
| AF | Multivariable | 1.76 | 0.86–3.59 | 0.123 | C=0.657; PH p=0.159 |

PH: Proportional Hazards, AF atrial fibrillation

Interaction (NT-proBNP×AF): p = 0.565.

**Panel B.** Procedural period early versus contemporary procedural periods

| **Period** | **Model** | **Hazard Ratio** | **95% CI** | **p-value** | **Harrell’s C; PH global p** |
| --- | --- | --- | --- | --- | --- |
| Early period | Univariable | 4.08 | 1.58–10.57 | 0.004 | — |
| Early period | Multivariable | 4.74 | 1.51–14.88 | 0.008 | C=0.768; PH p=0.391 |
| Contemporary period | Univariable | 1.91 | 1.23–2.95 | 0.004 | — |
| Contemporary period | Multivariable | 1.59 | 0.98–2.58 | 0.060 | C=0.660; PH p=0.598 |

Early period (2009–2016), Contemporary period: 2017–2023.

Interaction (NT-proBNP×period )= p= 0.082.

**Supplementary Table S4.** Multivariable Cox regression analysis for 2-year all-cause death or HF rehospitalization including Euroscore II (Model B)

| **Variable** | **Hazard Ratio** | **95% CI** | **p-value** |
| --- | --- | --- | --- |
| High NT-proBNP at admission | 1.88 | 1.22 – 2.89 | 0.004 |
| EuroSCORE II | 1.03 | 1.00 – 1.07 | 0.077 |
| Atrial Fibrillation | 1.36 | 0.88 – 2.11 | 0.165 |
| BMI (per unit) | 1.00 | 0.95 – 1.04 | 0.851 |
| Anemia | 1.31 | 0.87 – 1.99 | 0.199 |
| Smoking | 1.76 | 1.10 – 2.83 | 0.019 |
| Dyslipidemia | 0.72 | 0.48 – 1.09 | 0.118 |

*Abbreviations: CI, confidence interval. .EuroSCORE II scaled per 1% absolute risk,* NT-proBNP: N-terminal pro–B-type natriuretic peptide*, BMI, body mass index.*

**Supplementary Table S5.** Multivariable Cox regression for 2-year all-cause death or HF rehospitalization according to NT-proBNP trajectories including Euroscore II (Model B)

| **Variable** | **Hazard Ratio** | **95% CI** | **p-value** |
| --- | --- | --- | --- |
| NT-proBNP reduction | 1.12 | 0.44 – 2.83 | 0.809 |
| NT-proBNP increase | 2.50 | 1.08 – 5.79 | 0.033 |
| Persistently high NT-proBNP | 2.07 | 1.16 – 3.69 | 0.014 |
| EuroSCORE II (per 1% absolute risk) | 1.05 | 1.00 – 1.10 | 0.041 |
| BMI (per unit) | 0.99 | 0.94 – 1.05 | 0.852 |
| Anemia | 1.46 | 0.89 – 2.42 | 0.137 |
| Dyslipidemia | 0.66 | 0.40 – 1.06 | 0.088 |
| Atrial fibrillation | 1.25 | 0.76 – 2.07 | 0.385 |

*Abbreviations: CI, confidence interval,* NT-proBNP, N-terminal pro–B-type natriuretic peptide*, BMI, Body mass index.*
